# Clinical outcomes of chondroblastoma treated using synthetic bone substitute: risk factors for developing radiographic joint degeneration

**DOI:** 10.1186/s12957-020-01829-4

**Published:** 2020-03-02

**Authors:** Hidetatsu Outani, Shigeki Kakunaga, Kenichiro Hamada, Satoshi Takenaka, Sho Nakai, Naohiro Yasuda, Yoshinori Imura, Norifumi Naka, Nobuhito Araki, Takafumi Ueda, Hideki Yoshikawa

**Affiliations:** 1 Department of Orthopaedic Surgery, Osaka University Graduate School of Medicine, 2-2, Yamadaoka, Suita, Osaka, 565-0871 Japan; 2 Department of Orthopaedic Surgery, National Hospital Organization Osaka National Hospital, 2-1-14 Hoenzaka, Chuo-ku, Osaka, 540-0006 Japan; 3 Musculoskeletal Oncology Service, Osaka International Cancer Institute, 3-1-69 Otemae, Chuo-ku, Osaka, 541-8567 Japan; 4 Department of Orthopaedic Surgery, Ashiya Municipal Hospital, 39-1 Asahigaoka-cho, Ashiya, Hyogo 659-8502 Japan

**Keywords:** Chondroblastoma, Joint degeneration, Curettage

## Abstract

**Background:**

Chondroblastoma (CB) is a rare locally aggressive bone tumor that commonly occurs in the epiphysis or apophysis of long bones. Although surgical treatment of CB carries potential risk for physeal or articular cartilage damage, risk factors for joint degeneration have not been well described. In addition, we have mainly used synthetic bone substitute (SBS) to fill the bone defect after intralesional curettage as treatment for CB. This study thus aimed to evaluate the incidence of and risk factors for adjacent-joint radiographic degeneration after SBS treatment for CB.

**Methods:**

We retrospectively reviewed 48 patients treated for CB at our institutions between 1996 and 2017. Clinical data, radiographic images, treatments, and local recurrence were analyzed.

**Results:**

We identified 40 patients [29 males and 11 females with a mean age of 19 years (range, 8–35 years)] who received SBS to fill the defect after curettage with a minimum follow-up of 1 year. The mean follow-up period was 71 months (range, 13–239 months). A total of 8 patients (20%) developed local recurrence. Radiographic analysis showed that 5 patients (16.7%) developed radiographic joint degeneration. Joint degeneration was significantly associated with the affected joint (*p* = 0.004).

**Conclusions:**

Curettage and SBS filling had been found to be a reasonable treatment method for CB, which commonly occurs in the epiphysis or apophysis. Radiographic joint degeneration was not uncommon after CB treatment, especially in the talus and proximal humerus.

## Background

Chondroblastoma (CB) is a locally aggressive benign cartilaginous tumor that commonly occurs in childhood and adolescence [1]. CB accounts for approximately 1% of all primary bone tumors [2] and often affects the epiphysis or apophysis of long bones but can also originate from flat or short tubular bones [3, 4]. Studies have shown that most of the patients suffer from pain and limited motion in the adjacent joint [2, 3, 5]. Surgical treatment still remains the standard treatment for CB despite recent reports showing favorable results using radiofrequency ablation in selected patients [6, 7]. Surgical treatment often consists of meticulous curettage, either alone or combined with adjuvant therapy, followed by filling of the bone defect [2, 8–10]. Aggressive curettage may lead to the development of joint degeneration due to articular cartilage or physeal damage [1, 2, 8]. However, risk factors for developing joint degeneration after treatment of CB have not been well described. Furthermore, while bone grafting or cementation has been commonly used, we have preferred to use synthetic bone substitute (SBS) to fill the defect [2, 8]. Reports have shown SBS to be a useful and safe bone substitute for the treatment of benign bone tumors given that it can be well incorporated into the host bone without any allergic and neoplastic complications and prevent donor site complications of autogenous bone grafts, such as pain, infection, and nerve damage [11–13]. Considering that the effect of SBS on adjacent joint has not been also well described, this study aimed to investigate the risk factors for local recurrence and joint degenerative changes after CB treatment using SBS.

## Methods

We identified 48 patients histologically confirmed to have CB from our institutional database between 1996 and 2017. Among the 48 patients, four with less than 1 year of follow-up and one who underwent en bloc excision were excluded. Three patients who underwent curettage and cementation or had no packing were also excluded. Among the remaining 40 patients, four received adjuvant therapy (two with high-speed burr, one with distilled water, and one with phenol). The other patients underwent meticulous curettage alone, subsequently filling the defects with SBS. All SBSs used herein were made of hydroxyapatite ceramic. Imaging studies upon presentation, including radiographs, computed tomography (CT), and magnetic resonance imaging (MRI), were reviewed for lesion site, tumor size, and relation to the physis. The greatest dimension of the tumor was considered as the tumor size. Physeal involvement was indicated when tumors extended across the physis. Local recurrence was confirmed through MRI. Degenerative changes on radiographic analyses were classified using the Kellgren–Lawrence grading system [14]. All patients were followed up at our outpatient clinic at 3-month intervals for 2 years and then at 6-month intervals until 5 years. Radiographs were obtained at each visit, while local MRIs were obtained for any recurrence of symptoms or abnormal radiographic findings. Statistical analyses were performed using the SPSS 23.0 software (IBM Corp., Armonk, NY, USA). Local recurrence-free survival (LRFS) was determined using the Kaplan–Meier method with 95% confidence intervals (CIs). Univariate analysis using the log-rank test was used to compare the variables, while Pearson’s chi-squared test was used to evaluate the association between variables. A *p* value of ≤ 0.05 indicated a significant difference.

## Results

**Fig. 1:**
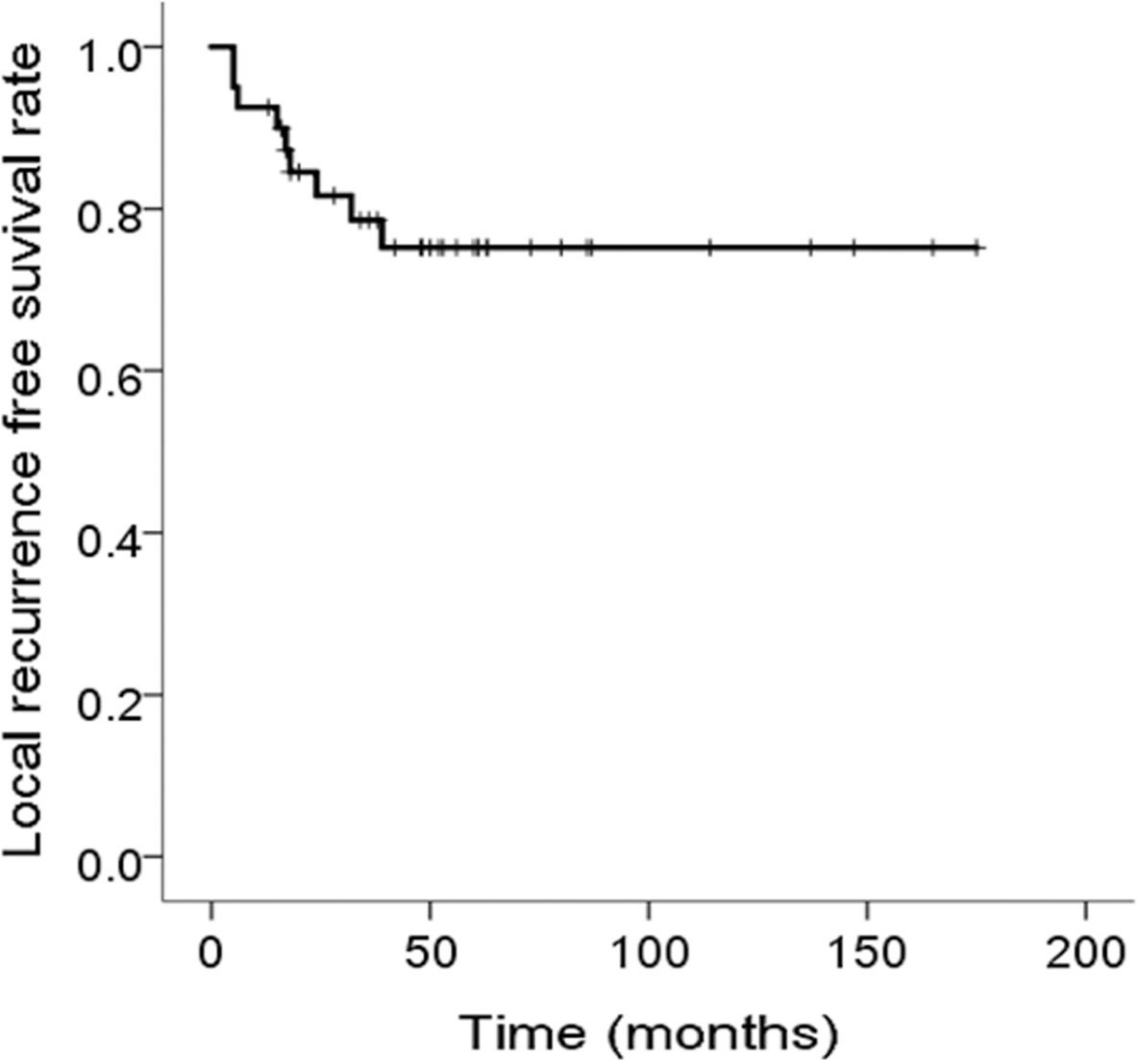
Kaplan–Meier curve for local recurrence-free survival among the 40 patients with chondroblastoma treated with synthetic bone substitute

A total of 40 patients with CB (29 male and 11 female patients) underwent curettage followed by SBS filling with a minimum follow-up of 1 year. The mean age at presentation was 19 (range, 8–35) years, while the mean follow-up duration was 71 (range, 13–239) months. Patient, tumor, and treatment characteristics are summarized in Table 1. Seven patients were < 14 years old, while 33 were ≥ 14 years old. The most common tumor site was the proximal femur (10, 25%), followed by the proximal tibia (7, 17.5%), calcaneus (7, 17.5%), distal femur (4, 10%), proximal humerus (4, 10%), patella (4, 10%), talus (2, 5%), distal humerus (1, 2.5%), and ischium (1, 2.5%). A total of 30 tumors developed in or spread into a subchondral bone were classified according to their adjacent joints (Table 1), while the other 10 were not adjacent to a joint. Physis involvement was observed in 6 patients (15%). The mean tumor size was 33 (range, 14–77) mm. Moreover, 17 tumors were < 30 mm, while 21 were ≥ 30 mm (two were unknown). One patient developed an extraosseus lesion. Seven patients underwent curettage using the intra-articular approach, while the remaining 33 patients underwent curettage using the extra-articular approach. Local recurrence occurred in 8 patients (20%) at a mean duration of 16 (range, 5–39) months. LRFS rate was 84.6% at 2 years (95% CI, 96.0–73.2) and 78.1% at 5 years (95% CI, 91.6–64.6) (Fig. 1). All 8 patients underwent a second curettage and SBS filling, among whom 7 had no further recurrences. One patient underwent a third curettage and SBS filing, after which no further recurrence occurred. Details of patients who developed local recurrence are summarized in Table 2. Three patients developed local complications, among whom one had limited range of motion in the affected shoulder joint, one had continuous scar pain, and one had femoral head necrosis after a second curettage of a trochanteric to neck lesion combined with compression hip screw fixation. None of the patients developed leg length discrepancy. All patients had adjacent joint preservation, while no patient received amputation or joint replacement. Univariate analysis could not identify any significant risk factors for local recurrence probably due to the small sample size (Table 1).

**Table 1:** Association between local recurrence or joint degeneration and clinical factors

Variables		Patient number	Local recurrence			Degenerative changes		
			(−)	(+)	Log-rank *P*	(−)	(+)	Chi-squared *P*
Sex	Male	29	23	6	0.791	19	3	0.854
	Female	11	9	2		6	2	
Age	< 14	7	6	1	0.661	3	2	0.381
	≥ 14	33	26	7		22	3	
Size	< 30 mm	17	13	4	0.175	14	2	0.87
	≥ 30 mm	21	19	2		11	3	
Physeal involvement	Yes	6	5	1	0.846	4	2	0.254
	No	34	27	7		21	3	
Surgical approach	Intra-articular	7	7	0	0.182	7	0	0.44
	Extra-articular	33	25	8		18	5	
Affected joint	Knee	15	13	2	0.401	15	0	**0.004**
	Hip	5	5	0		5	0	
	Shoulder	4	2	2		2	2	
	Subtalar	4	2	2		1	3	
	Elbow	1	1	0		1	0	
	Ankle	1	1	0		1	0	
	No	10	8	2				
Local recurrence	Yes	8				4	2	0.54
	No	32				21	3

**Table 2:** Details of patients with local recurrence

Case	Age/sex	Location	Time to LR (months)	Treatment of first LR	Result
1	12/F	Proximal humerus	15	Curettage and packing SBS	No further LR (NED 14 months)
2	15/M	Proximal tibia	6	Curettage and packing SBS	No further LR (NED 51 months)
3	31/M	Calcaneus	39	Curettage and packing SBS	No further LR (NED 40 months)
4	18/M	Proximal femur	5	Curettage and packing SBS and internal fixation	No further LR (NED 115 months)
5	18/M	Proximal femur	32	Curettage and packing SBS and internal fixation	No further LR, screw cutout, and implant removal (NED 207 months)
6	15/M	Proximal tibia	5	Curettage and packing SBS	No further LR (NED 63 months)
7	17/M	Proximal humerus	17	Curettage and packing SBS	Second LR after 12 months, further curettage, and packing SBS (NED 21 months)
8	22/M	Calcaneus	18	Curettage and packing SBS	No further LR (NED 43 months)

*SBS* synthetic bone substitute, *LR* local recurrence, *NED* no evidence of disease

**Fig. 2:**
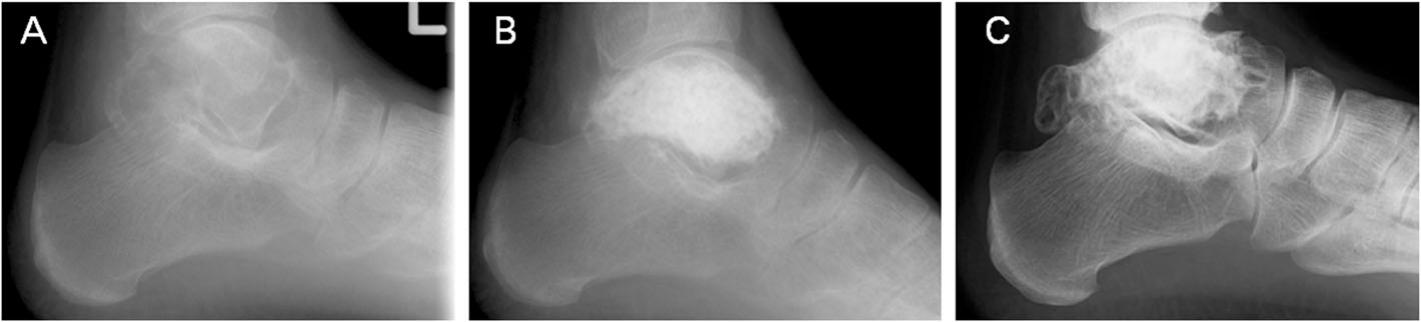
Chondroblastoma arising from the left talus. **a** Plain radiography upon referral showing a lytic lesion in the talus. **b** One month after surgery. **c** Fourteen years after surgery

**Fig. 3:**
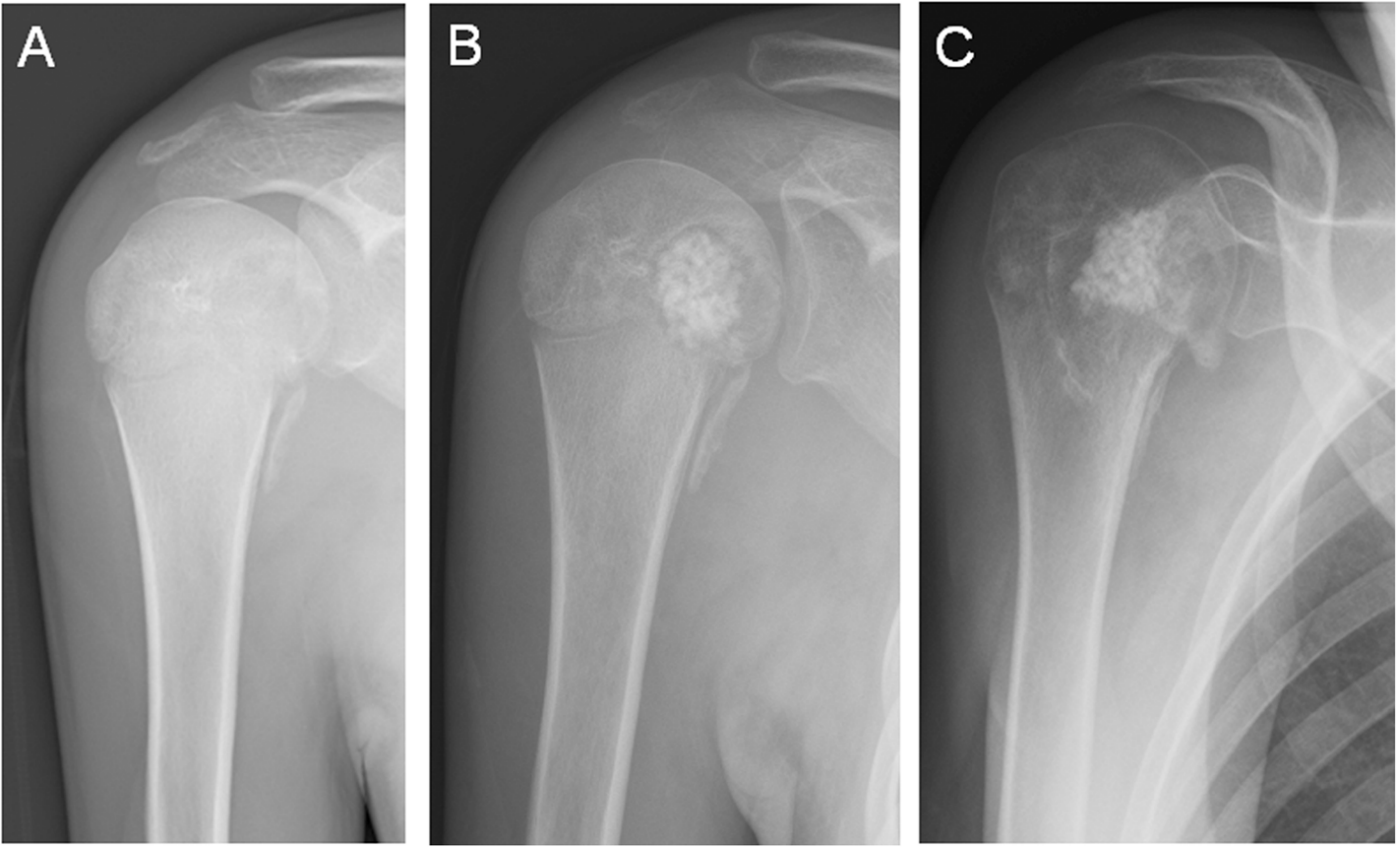
Chondroblastoma arising from the right proximal humerus. **a** Plain radiography upon referral showing a lytic lesion in the proximal humerus. **b** One month after surgery. **c** One and a half year after surgery. This patient had mid shoulder pain

Radiographs at the final follow-up showed that among the 30 patients whose tumor occurred adjacent to a joint lesion, 5 (16.7 %) exhibited joint degeneration (two cases were grade I, one grade II, and two grade III) (Fig. 2). Among the five patients, one had mild shoulder pain (Fig. 3), while the others were asymptomatic in the affected joint. Degenerative changes were significantly associated with the affected joint (*p* = 0.004) (Table 1). Among the joint groups, the shoulder and subtalar groups had greater correlation with degenerative changes compared to the other groups.

## Discussion

Considering that CB is essentially a benign bone tumor that rarely metastasizes [2, 3, 8, 15], intralesional curettage followed by bone defect filling has been considered the standard treatment. The predominance of epiphyseal occurrence has made aggressive curettage challenging to perform due to concerns regarding physeal damage, which has been considered to be associated with high local recurrence rates [1, 2]. Several studies have reported a local recurrence rates ranging from 3 to 32% [1–3, 5, 8, 15–18], which have been consistent with results presented herein. Moreover, we do not believe that using SBS for bone defect filling caused high local recurrence rates but instead that less aggressive curettage in an attempt to prevent physeal or articular cartilage damage may have caused local recurrence. To compensate for incomplete curettage, some authors have recommended the use of adjuvant therapy, such as cryosurgery [9, 10]. However, the role of adjuvant therapy, including high-speed burr, has not been well elucidated. Given the high risk for local recurrence, further study of adjuvant therapy is desired. Although age, location (pelvis, proximal femur, and proximal humerus), epiphyseal CB, and previous surgery have been identified as risk factors of local recurrence [1–3, 15, 18, 19], their significance has still remained controversial. Lin et al. stated that local recurrence seemed to be associated with inadequate surgery and the biological aggressiveness of the tumor [15]. Although we agree with this speculation, unfortunately, no reliable factors defining biological aggressiveness have been found [19]. While a component of aneurysmal bone cysts has been considered to be associated with aggressive CB [20], other studies have failed to confirm this [2, 15, 21]. Radiographic staging, which attempted to classify the aggressiveness of CB, also failed to show the correlation between biological aggressiveness and local recurrence [2, 5]. Moreover, studies have failed to identify physeal status as a factor associated with aggressive CB [1, 2, 15]. In this study, physeal involvement of CB was not associated with a local recurrence. Similarly, Sailhan et al. observed that epiphyseal–metaphyseal CB was not associated with a higher recurrence rate compared to epiphyseal CB [1]. Furthermore, no studies have succeeded to identify predictive pathological features for aggressive CB [19, 21].

Typically, autogenous or allogeneic bone graft or polymethylmethacrylate has been used to fill the bone defect after curettage of benign bone tumor. However, we have preferred to use SBS given that it can provide a sufficient amount of bone substitutes despite large defects and prevent donor site morbidity. Accordingly, Uchida et al. demonstrated good bone ingrowth into the SBS after benign bone tumor curettage and concluded that SBS was a safe and convenient implant material that aided bone defect regeneration [11]. Yamamoto et al. had reported similar findings, while Matsumine et al. demonstrated good long-term outcomes following SBS implantation after bone tumor surgery [12, 13]. However, the effects of SBS on the adjacent joint have not been well described. Yamamoto et al. reported five cases in which SBS had been successfully implanted into subchondral bone lesions without any joint degeneration [12]. Moreover, Matsumine et al. reported one patient with a giant cell tumor of the proximal tibia who developed radiographic joint degeneration 8 years after SBS implantation [13]. The aforementioned study found that SBS incorporation was associated with neither epiphyseal location nor cavity volume but was significantly associated with males and younger age [13]. To our knowledge, the present study has been the largest series on SBS implantation into subchondral lesions after benign tumor surgery. Among the 30 patients who had undergone SBS implantation into subchondral lesion, 5 (16.7%) showed radiographic degeneration in the affected joint, although most of them were asymptomatic.

Little information has been available regarding joint degeneration after curettage for epiphyseal CB [1, 8, 17, 22]. Farfalli et al. reported that 20 of 53 patients with epiphyseal CB (38%) developed secondary osteoarthritis with a mean follow-up of 77 months and a 5- and 10-year joint survival of 90% and 74%, respectively [17]. Moreover, Sailhan et al. found that 8% of patients with CB showed poor functional outcomes determined using radiographic findings of arthritis [1]. Our results showed that 16.7% of tumors adjacent to the joint showed radiographic degenerative changes, while 3.3% (1 of 30 patients) exhibited slight pain around the joint following SBS use. In contrast with the high incidence of secondary osteoarthritis reported by Farfalli et al., our results showed that SBS seemed to be safe even for patients with epiphyseal CB. In addition, our findings revealed that the shoulder and subtalar joints had a tendency to develop radiographic degenerative changes. However, this result should be interpreted with caution due to the small number of patients. Nonetheless, Suneja et al. reported that the talus and proximal humerus were associated with worse functional results [2], while Farfalli et al. found that secondary osteoarthritis appeared to be more common among patients with hip and talus CB [17]. Collectively, although SBS can be safely used for subchondral lesions, awareness of the increased risk for developing joint degeneration after CB treatment is needed. Moreover, patients need to be informed about the risk for joint arthritis, especially for those with tumors of talus and proximal humerus.

The present study had several limitations worth considering. First, the small number of patients and varying tumor locations weakened the statistical power of the current study. Second, the relatively short follow-up periods resulted in lower estimation of developing degenerative changes. Third, no control group had been included, and we could not draw any definitive conclusion regarding the effect of SBS on the adjacent joint.

## Conclusions

The findings obtained herein showed that SBS can be used safely even in the treatment of epiphyseal CB. Moreover, radiographic joint degeneration was not uncommon after intralesional curettage of CB, especially in the talus and proximal humerus.

## Data Availability

The dataset for this study is not publicly available but can be made available upon reasonable request.
